# Supplementary material for: Lutein/Zeaxanthin Isomers and Quercetagetin Combination Safeguards the Retina from Photo-Oxidative Damage by Modulating Neuroplasticity Markers and the Nrf2 Pathway
Source: Pharmaceuticals (Basel). 2023 Nov 1;16(11):1543. doi: 10.3390/ph16111543 (PMC10675275; doi:10.3390/ph16111543)
Supplement: Supplementary file 1 [file pharmaceuticals-16-01543-s001.zip › pharmaceuticals-2519535-supplementary.pdf]

Supplementary Tables for pairwise comparisons

Supplementary Table S1. The pairwise comparison of serum malondialdehyde (MDA), superoxide dismutase (SOD), catalase (CAT), and glutathione peroxidase (GPX) levels between groups in LED-induced retinal damage in rats.

Parameters	Pairwise comparison	P value
**MDA**	LED+QC vs. LED+QCG2	P > 0.05 (NS)
	LED+QC vs. LED+QCG1	P < 0.05
	LED+L/Z+QCG (1:0.5) vs. LED+L/Z+QCG (0.5:0.5)	P < 0.01
	LED+L/Z vs. LED+QCG2<br>LED+QCG1 vs. LED+QCG2	P < 0.001
	Other pairwise comparisons	P < 0.0001
**SOD**	LED+L/Z vs. LED+QC<br>LED+L/Z vs. LED+QCG2<br>LED+QC vs. LED+QCG1<br>LED+QC vs. LED+QCG2<br>LED+L/Z+QCG (1:0.5) vs. LED+L/Z+QCG (0.5:0.5)	P > 0.05 (NS)
	LED+L/Z vs. LED+QCG1<br>LED+L/Z vs. LED+L/Z+QCG (0.5:0.5)<br>LED+QCG1 vs. LED+QCG2<br>LED+L/Z+QCG (1:1) vs. LED+L/Z+QCG (1:0.5)	P < 0.05
	LED+QCG2 vs. LED+L/Z+QCG (0.5:0.5)	P < 0.01
	LED+QC vs. LED+L/Z+QCG (0.5:0.5)	P < 0.001
	Other pairwise comparisons	P < 0.0001
**CAT**	LED+L/Z vs. LED+QC<br>LED+L/Z vs. LED+QCG1<br>LED+L/Z vs. LED+QCG2<br>LED+L/Z vs. LED+L/Z+QCG (0.5:0.5)<br>LED+QC vs. LED+QCG1<br>LED+QC vs. LED+QCG2<br>LED+QCG2 vs. LED+L/Z+QCG (0.5:0.5)<br>LED+L/Z+QCG (1:0.5) vs. LED+L/Z+QCG (0.5:0.5)	P > 0.05 (NS)
	LED+QCG1 vs. LED+QCG2<br>LED+QCG2 vs. LED+L/Z+QCG (1:0.5)	P < 0.05
	LED+L/Z vs. LED+L/Z+QCG (1:0.5)<br>LED+L/Z+QCG (1:1) vs. LED+L/Z+QCG (1:0.5)	P < 0.01
	LED+QC vs. LED+L/Z+QCG (0.5:0.5)	P < 0.001
	Other pairwise comparisons	P < 0.0001
**GPX**	LED+L/Z vs. LED+QC<br>LED+L/Z vs. LED+QCG2<br>LED+L/Z vs. LED+L/Z+QCG (1:0.5)<br>LED+L/Z vs. LED+L/Z+QCG (0.5:0.5)<br>LED+QC vs. LED+QCG1<br>LED+QC vs. LED+QCG2<br>LED+QC vs. LED+L/Z+QCG (0.5:0.5)<br>LED+QCG2 vs. LED+L/Z+QCG (0.5:0.5)<br>LED+L/Z+QCG (1:0.5) vs. LED+L/Z+QCG (0.5:0.5)<br>LED+QCG2 vs. LED+L/Z+QCG (1:0.5)	P > 0.05 (NS)
	LED+L/Z vs. LED+QCG1<br>LED+QCG1 vs. LED+QCG2	P < 0.05
	LED+QC vs. LED+L/Z+QCG (1:0.5)<br>LED+QCG1 vs. LED+L/Z+QCG (0.5:0.5)<br>LED+L/Z+QCG (1:1) vs. LED+L/Z+QCG (1:0.5)	P < 0.001
	Other pairwise comparisons	P < 0.0001

n=7, ANOVA and Bonferroni's multiple comparisons test. NS: not significant

Supplementary Table S2. The pairwise comparison of serum interleukin-1β (IL-1β), IL-6, and tumor necrosis factor-α (TNF-α) levels between groups in LED-induced retinal damage in rats.

Parameters	Pairwise comparison	P value
**IL-1β**	LED+L/Z vs. LED+QC	P > 0.05 (NS)
	LED+L/Z vs. LED+QCG1	
	LED+L/Z vs. LED+QCG2	
	LED+QC vs. LED+QCG1	
	LED+QCG1 vs. LED+QCG2	
	LED+L/Z+QCG (1:1) vs. LED+L/Z+QCG (1:0.5)	
	LED+L/Z+QCG (1:0.5) vs. LED+L/Z+QCG (0.5:0.5)	
	LED+QC vs. LED+QCG2	P < 0.01
	LED+QCG2 vs. LED+L/Z+QCG (0.5:0.5)	
	Other pairwise comparisons	P < 0.0001
**IL-6**	LED+L/Z vs. LED+QC	P > 0.05 (NS)
	LED+L/Z vs. LED+QCG1	
	LED+L/Z vs. LED+QCG2	
	LED+L/Z vs. LED+L/Z+QCG (1:0.5)	
	LED+L/Z vs. LED+L/Z+QCG (0.5:0.5)	
	LED+QC vs. LED+QCG1	
	LED+QCG1 vs. LED+QCG2	
	LED+QCG2 vs. LED+L/Z+QCG (1:0.5)	
	LED+QCG2 vs. LED+L/Z+QCG (0.5:0.5)	
	LED+L/Z+QCG (1:0.5) vs. LED+L/Z+QCG (0.5:0.5)	
	LED+QC vs. LED+QCG2	P < 0.01
	LED+QCG1 vs. LED+L/Z+QCG (0.5:0.5)	
	LED+QC vs. LED+L/Z+QCG (0.5:0.5)	P < 0.001
	LED+QCG1 vs. LED+L/Z+QCG (1:0.5)	
	Other pairwise comparisons	P < 0.0001
**TNF-α**	LED+L/Z vs. LED+QC	P > 0.05 (NS)
	LED+L/Z vs. LED+QCG1	
	LED+L/Z vs. LED+QCG2	
	LED+QC vs. LED+QCG1	
	LED+L/Z+QCG (1:1) vs. LED+L/Z+QCG (1:0.5)	
	LED+L/Z+QCG (1:0.5) vs. LED+L/Z+QCG (0.5:0.5)	
	LED+QC vs. LED+QCG2	P < 0.01
	LED+QCG1 vs. LED+QCG2	
	LED+L/Z+QCG (1:1) vs. LED+L/Z+QCG (0.5:0.5)	P < 0.001
	Other pairwise comparisons	P < 0.0001

n=7, ANOVA and Bonferroni's multiple comparisons test. NS: not significant

Supplementary Table S3. The pairwise comparison of retinal Bcl-2-associated X protein (Bax), B-cell lymphoma 2 (Bcl-2), and cyste-ine-aspartic acid protease-3 (Caspase-3) levels between groups in LED-induced retinal damage in rats.

Parameters	Pairwise comparison	P value
**Bax**	LED vs. LED+QCG1<br>LED+L/Z vs. LED+QCG2<br>LED+L/Z vs. LED+L/Z+QCG (0.5:0.5)<br>LED+QC vs. LED+QCG2<br>LED+QCG2 vs. LED+L/Z+QCG (0.5:0.5)	P > 0.05 (NS)
	LED+L/Z vs. LED+QC<br>LED+QC vs. LED+QCG1<br>LED+QCG1 vs. LED+QCG2<br>LED+L/Z+QCG (1:1) vs. LED+L/Z+QCG (1:0.5)	P < 0.01
	LED+QC vs. LED+L/Z+QCG (0.5:0.5)<br>LED+L/Z+QCG (1:0.5) vs. LED+L/Z+QCG (0.5:0.5)	P < 0.001
	Other pairwise comparisons	P < 0.0001
**Bcl-2**	LED+L/Z vs. LED+QC<br>LED+QC vs. LED+QCG2	P > 0.05 (NS)
	LED+L/Z vs. LED+L/Z+QCG (0.5:0.5)	P < 0.05
	LED+L/Z vs. LED+QCG2<br>LED+QC vs. LED+QCG1	P < 0.01
	LED+L/Z+QCG (1:1) vs. LED+L/Z+QCG (1:0.5)	P < 0.001
	Other pairwise comparisons	P < 0.0001
**Caspase-3**	LED+L/Z vs. LED+QC<br>LED+L/Z vs. LED+L/Z+QCG (1:0.5)<br>LED+L/Z vs. LED+L/Z+QCG (0.5:0.5)<br>LED+QC vs. LED+L/Z+QCG (0.5:0.5)<br>LED+QCG1 vs. LED+QCG2<br>LED+L/Z+QCG (1:0.5) vs. LED+L/Z+QCG (0.5:0.5)	P > 0.05 (NS)
	LED+QC vs. LED+L/Z+QCG (1:0.5)<br>LED+L/Z+QCG (1:1) vs. LED+L/Z+QCG (1:0.5)	P < 0.05
	Other pairwise comparisons	P < 0.0001

n=7, ANOVA and Bonferroni's multiple comparisons test. NS: not significant

Supplementary Table S4. The pairwise comparison of retinal nuclear factor erythroid 2-related factor 2 (Nrf2), heme oxygenase-1 (HO-1), and nuclear factor kappa B p65 subunit (NF-κB p65) levels between groups in LED-induced retinal damage in rats.

Parameters	Pairwise comparison	P value
**Nrf2**	LED+L/Z vs. LED+QC<br>LED+QC vs. LED+QCG2<br>LED+L/Z+QCG (1:0.5) vs. LED+L/Z+QCG (0.5:0.5)	P > 0.05
	LED+L/Z vs. LED+QCG1<br>LED+L/Z vs. LED+QCG2<br>LED+QCG2 vs. LED+L/Z+QCG (0.5:0.5)	P < 0.05
	LED vs. LED+QCG1	P < 0.01
	LED+QC vs. LED+QCG1<br>LED+L/Z+QCG (1:1) vs. LED+L/Z+QCG (1:0.5)	P < 0.001
	Other pairwise comparisons	P < 0.0001
**HO-1**	LED+QC vs. LED+QCG2<br>LED+L/Z+QCG (1:0.5) vs. LED+L/Z+QCG (0.5:0.5)	P > 0.05<br>(NS)
	LED+L/Z vs. LED+QC<br>LED+L/Z vs. LED+L/Z+QCG (0.5:0.5)	P < 0.01
	Other pairwise comparisons	P < 0.0001
**NF-κB p65**	LED+L/Z vs. LED+QC<br>LED+L/Z vs. LED+QCG2<br>LED+QC vs. LED+QCG1<br>LED+QC vs. LED+QCG2<br>LED+QCG1 vs. LED+QCG2<br>LED+L/Z+QCG (1:1) vs. LED+L/Z+QCG (1:0.5)	P > 0.05<br>(NS)
	LED+L/Z/Z vs. LED+QCG1	P < 0.05
	LED+L/Z/Z+QCG (1:0.5) vs. LED+L/Z+QCG (0.5:0.5)	P < 0.01
	LED+L/Z vs. LED+L/Z+QCG (0.5:0.5)	P < 0.001
	Other pairwise comparisons	P < 0.0001

n=7, ANOVA and Bonferroni's multiple comparisons test. NS: not significant

Supplementary Table S5. The pairwise comparison of retinal intercellular adhesion molecule-1 (ICAM), growth-associated protein-43 (GAP43), glial fibrillary acid protein (GFAP), neural cell adhesion molecule (NCAM), and monocyte chemoattractant protein 1 (MCP-1) levels between groups in LED-induced retinal damage in rats.

Parameters	Pairwise comparison	P value
**ICAM**	LED+QC vs. LED+QCG2	P > 0.05 (NS)
	LED+L/Z vs. LED+L/Z+QCG (0.5:0.5)	P < 0.01
	Control vs. LED+L/Z+QCG (1:1)	P < 0.001
	Other pairwise comparisons	P < 0.0001
**GAP43**	LED+QC vs. LED+QCG2<br>LED+L/Z+QCG (1:0.5) vs. LED+L/Z+QCG (0.5:0.5)	P > 0.05 (NS)
	Other pairwise comparisons	P < 0.0001
**GFAP**	LED+L/Z vs. LED+QC<br>LED+L/Z vs. LED+QCG2<br>LED+QC vs. LED+QCG2<br>LED+L/Z+QCG (1:1) vs. LED+L/Z+QCG (1:0.5)	P > 0.05 (NS)
	LED+L/Z vs. LED+L/Z+QCG (0.5:0.5)	P < 0.05
	LED+QCG2 vs. LED+L/Z+QCG (0.5:0.5)	P < 0.01
	Other pairwise comparisons	P < 0.0001
**NCAM**	LED+QCG1 vs. LED+QCG2<br>LED+L/Z+QCG (1:0.5) vs. LED+L/Z+QCG (0.5:0.5)	P < 0.01
	Other pairwise comparisons	P < 0.0001
**MCP-1**	LED+L/Z vs. LED+QCG2	P > 0.05 (NS)
	LED+L/Z+QCG (1:0.5) vs. LED+L/Z+QCG (0.5:0.5)	P < 0.001
	Other pairwise comparisons	P < 0.0001

n=7, ANOVA and Bonferroni's multiple comparisons test. NS: not significant

Supplementary Table S6. The pairwise comparison of outer nuclear layer (ONL) thickness level between groups in LED-induced retinal damage in rats.

Parameters	Pairwise comparison	P value
**ICAM**	LED vs. LED+QC<br>LED vs. LED+QCG1<br>LED+L/Z vs. LED+QC<br>LED+L/Z vs. LED+QCG1<br>LED+L/Z vs. LED+QCG2<br>LED+L/Z vs. LED+L/Z+QCG (0.5:0.5)<br>LED+QC vs. LED+QCG1<br>LED+QC vs. LED+QCG2<br>LED+QCG1 vs. LED+QCG2<br>LED+QCG2 vs. LED+L/Z+QCG (0.5:0.5)<br>LED+L/Z+QCG (1:1) vs. LED+L/Z+QCG (1:0.5)<br>LED+L/Z+QCG (1:1) vs. LED+L/Z+QCG (0.5:0.5)<br>LED+L/Z+QCG (1:0.5) vs. LED+L/Z+QCG (0.5:0.5)	P > 0.05 (NS)
	Control vs. LED+L/Z+QCG (1:1)<br>Control vs. LED+L/Z+QCG (1:0.5)<br>LED vs. LED+L/Z, LED vs. LED+QCG2<br>LED+QC vs. LED+L/Z+QCG (0.5:0.5)	P < 0.01
	LED+L/Z vs. LED+L/Z+QCG (1:0.5)<br>LED+QCG1 vs. LED+L/Z+QCG (0.5:0.5)	P < 0.001
	Other pairwise comparisons	P < 0.0001

n=7, ANOVA and Bonferroni's multiple comparisons test. NS: not significant
